# Abnormalities of White Matter Microstructure in Unmedicated Obsessive-Compulsive Disorder and Changes after Medication

**DOI:** 10.1371/journal.pone.0035889

**Published:** 2012-04-27

**Authors:** Qing Fan, Xu Yan, Jijun Wang, Ying Chen, Xuemei Wang, Chunbo Li, Ling Tan, Chao You, Tianhong Zhang, Sai Zuo, Dongrong Xu, Kemin Chen, Jodie Marie Finlayson-Burden, Zeping Xiao

**Affiliations:** 1 Shanghai Mental Health Center, School of Medicine, Shanghai Jiao Tong University, Shanghai, China; 2 Key Laboratory of Brain Functional Genomics, Ministry of Education, Shanghai Key Laboratory of Brain Functional Genomics, Shanghai Key Laboratory of Magnetic Resonance, East China Normal University, Shanghai, China; 3 Shanghai TCM-Intergrated Hospital, Shanghai, China; 4 Radiology Department, School of Medicine, Shanghai Jiao Tong University, Ruijin Hospital, Shanghai, China; 5 Nottingham Institute of Mental Health, University of Nottingham, Nottingham, England; The University of Melbourne, Australia

## Abstract

**Background:**

Abnormalities of myelin integrity have been reported in obsessive-compulsive disorder (OCD) using multi-parameter maps of diffusion tensor imaging (DTI). However, it was still unknown to what degree these abnormalities might be affected by pharmacological treatment.

**Objective:**

To investigate whether the abnormalities of white matter microstructure including myelin integrity exist in OCD and whether they are affected by medication.

**Methodology and Principal Findings:**

Parameter maps of DTI, including fractional anisotropy (FA), axial diffusivity (AD), radial diffusivity (RD) and mean diffusivity (MD), were acquired from 27 unmedicated OCD patients (including 13 drug-naïve individuals) and 23 healthy controls. Voxel-based analysis was then performed to detect regions with significant group difference. We compared the DTI-derived parameters of 15 patients before and after 12-week Selective Serotonin Reuptake Inhibitor (SSRI) therapies. Significant differences of DTI-derived parameters were observed between OCD and healthy groups in multiple structures, mainly within the fronto-striato-thalamo-cortical loop. An increased RD in combination with no change in AD among OCD patients was found in the left medial superior frontal gyrus, temporo-parietal lobe, occipital lobe, striatum, insula and right midbrain. There was no statistical difference in DTI-derived parameters between drug-naive and previously medicated OCD patients. After being medicated, OCD patients showed a reduction in RD of the left striatum and right midbrain, and in MD of the right midbrain.

**Conclusion:**

Our preliminary findings suggest that abnormalities of white matter microstructure, particularly in terms of myelin integrity, are primari ly located within the fronto-striato-thalamo-cortical circuit of individuals with OCD. Some abnormalities may be partly reversed by SSRI treatment.

## Introduction

Obsessive-compulsive disorder (OCD) is one of the most common mental disorders in the general population, with a prevalence rate of 2% to 3% [1]. The main clinical manifestations seen in OCD patients are recurrent, intrusive and distressing thoughts and/or repetitive behaviors, resulting in significantly impaired occupational and social functioning [2]. However, the neurobiological mechanisms of OCD are still unknown.

Previous structural and functional neuroimaging studies have supported the role of fronto-striato-thalamo-cortical circuit impairment in the pathogenesis of OCD. Rotge et al. [3] found volumetric differences between OCD patients and control subjects in the anterior cingulate cortex, orbitofrontal cortex and thalamus using a meta-analysis. Another meta-analysis, conducted by Whiteside et al. [4] using results from positron emission tomography (PET) and single photon emission computed tomography (SPECT), proved that significant differences of radiotracer uptake do exist between OCD patients and healthy controls in the orbital frontal cortex and caudate nucleus.

Diffusion tensor imaging (DTI) is a non-invasive method that maps the diffusivity of water molecules in tissue [5]. It is sensitive to the orientation and integrity of the underlying white matter fiber in vivo. Restricted by cell membrane and myelinlayers, the diffusivity perpendicular to fiber direction (radial diffusivity, RD) is lower than that along the length of the fiber (axial diffusivity, AD). Fractional anisotropy (FA) is the most commonly used index to detect this anisotropy, and is considered a sensitive marker of changes in tissue microstructures. Another index, mean diffusivity (MD), measures the average diffusivity in all directions, is frequently used as a complement to FA.

Several DTI studies have investigated white matter abnormalities in OCD patients relative to healthy subjects. These studies have generally supported abnormalities of the fronto-striato-thalamo-cortical loop in the pathogenesis of OCD. Within cortical structures, higher FA and apparent diffusion coefficient (ADC) were observed in the medial frontal region [6], [7], and lower FA was found in the parietal lobe [6], [8], left lingual gyrus and occipital lobe white matter [8]. In the cingulum, lower FA was reported in the anterior cingulated gyrus [8], right posterior cingulated gyrus [9] and cingulated bundle [10],while Cannistraro et al. [11] showed greater FA values in the left cingulum bundle and lower FA values in the right cingulum bundle. Yoo et al. [12] have reported higher FA in the corpus callosum, while Nakamae et al. [13] found smaller FA in the anterior body of the corpus callosum. Saito et al. [14] showed FA reduction in the rostrum of the corpus callosum and Garibotto et al. [10] also reported lower FA in the splenium of the corpus callosum and altered principal diffusion direction (PDD) along the corpus callosum. Greater FA measures were found in the left anterior limb of internal capsule [11], white matter around the right caudate [12] and bilateral semioval center [7]. However, the results of these studies were inconsistent and most of the studies used FA alone as a marker for studying white matter abnormality.

Recent debates have focused upon the interpretation of DTI parameters, suggesting that FA alone is insufficient for characterizing changes in tissue microstructure [15], [16]. Axial diffusivity and radial diffusivity have been proposed as a more specific markers for axonal and myelin injury, respectively [17]. Increased RD without significant changes in AD was found in the demyelinated mouse brain [18], [19]. In recent human studies, these parameters were used in combination with FA to investigate white matter microstructure in schizophrenia [20]–[24], depression [25] and autism [26], [27]. These studies found increased RD and insignificant changes in AD, suggesting deficits in myelination. However, a recent report has suggested caution in interpreting changes of the axial and radial diffusivities [28].

To our knowledge, only one OCD study has employed FA, RD and AD measures [29]. This study found myelin abnormalities represented by increased RD and normal AD in the body of the corpus callosum. However, this study had a cross-sectional design and 10 of the 21 OCD patients were receiving stable doses of drug treatments.

In the current study, we employed the DTI technique to study white matter abnormality in OCD patients using multiple parameters including FA, AD, RD and MD. These parameters were compared for patients before and after 12-week Selective Serotonin Reuptake Inhibitor (SSRI) pharmacotherapy. We hypothesized that abnormalities of white matter microstructure including myelin integrity would be found in OCD patients. We also predicted that sufficient medication would normalize the changes in white matter microstructure.

## Methods

### Ethics statement

After the study procedure had been explained to the participants, written informed consent was collected from all participants involved in the study. The research was approved by the local institutional ethics board (Institutional Review Board of Shanghai Mental Health Center, Shanghai Jiao Tong University School of Medicine).

### Participants

Twenty-seven patients with OCD and 23 healthy controls participated in the study. All participants were Chinese and right handed, and were generally matched for age, sex and education. The OCD patients were recruited from an outpatient clinic at Shanghai Mental Health Center, Shanghai, China. Inclusion criteria for patients with OCD were as follows: age between 18 and 54; no less than 9 years in education; a DSM-IV diagnosis of OCD; and being free of pharmacotherapy for at least 8 weeks before baseline (13 of the 27 OCD patients were drug-naive). Patients who presented with comorbid Axis I psychiatric disorders and a history of neurologic disorders were excluded. Two OCD patients who fulfilled inclusion criteria refused to participate in the MRI scanning. They were not counted within the OCD sample total of 27. Control participants were recruited through local print advertising. Inclusion criteria for the healthy controls were: age between 18 and 54; no less than 9 years in education; no DSM-IV Axis I diagnosis; and absence of a history of neurologic disorders.

A trained psychiatrist interviewed all participants with the Mini-International Neuropsychiatric Interview (M.I.N.I.) for DSM-IV (Diagnostic and Statistical Manual of Mental Disorders, 1994) Axis I mental disorders. The obsessive-compulsive symptoms of each participant were assessed using the Yale-Brown Obsessive Compulsive Scale (Y-BOCS). We applied the Hamilton Anxiety Rating Scale (HAMA, 14 items) and Hamilton Depression Rating Scale (HAMD, 24 items) to measure participants' anxiety and depression symptoms respectively.

There were no significant differences between the 27 patients and the 23 controls in age (t = −1.62, df = 48, p = 0.11), gender (χ^2^ = 0.03, df = 1, p = 0.87) or education (t = −0.62, df = 48, p = 0.52). OCD patients showed higher mean HAMA scores (t = 12.13, df = 48, p<0.001) and mean HAMD scores (t = 9.00, df = 48, p<0.001) than did the healthy controls (Table 1). There were no significant differences between the 13 drug-naive and 14 previously medicated OCD patients in terms of age (t = 0.42, df = 25, p = 0.68), gender (χ^2^ = 0.22, df = 1, p = 0.88), education (t = −0.52, df = 25, p = 0.61), age at onset (t = 0.93, df = 25, p = 0.36), illness duration (t = −0.53, df = 25, p = 0.61), mean Y-BOCS scores (t = 0.31, df = 25, p = 0.76), mean HAMA scores (t = 0.79, df = 25, p = 0.44) or mean HAMD scores (t = 1.35, df = 25, p = 0.19) at baseline (Table 1).

**Table 1:** The Demographics and Clinical Characteristics of the Baseline Participants.

Variable	OCD whole group (n = 27)	Drug-naïve OCD subgroup (n = 13)	previously medicated OCD subgroup (n = 14)	Control group (n = 23)
Age, mean± SD, y	25.5±7.0	26.1±9.3	24.9±4.1	28.8±7.6
Sex, No. M/F	17/10	8/5	9/5	15/8
Education, mean ± SD, y	14.0±2.9	13.7±3.1	14.3±2.8	14.6±3.7
age at onset, mean ± SD, y	20.4±7.9	21.9±10.1	19.1±5.2	-
illness duration, mean ± SD, y	4.8±4.0	4.3±4.6	5.1±3.6	-
Total YBOCS score, mean ± SD	22.0±4.9	22.3±4.7	21.7±5.4	0
Obsessive subscale	13.3±3.0	13.4±3.3	13.2±2.9	0
Compulsive subscale	8.7±4.5	8.9±4.3	8.5±4.8	0
HAMA score, mean ± SD	12.8±4.3	13.5±5.0	12.1±3.6	1.0±1.8
HAMD score, mean ± SD	12.4±5.4	13.9±6.1	11.1±4.5	1.4±2.3

### Pharmacotherapy

After receiving clinical feature measurements and DTI scans at baseline, 15 of the 27 OCD patients agreed to accept SSRI treatment only (6 of 15 OCD patients were drug-naive). The decision as to the specific SSRI drug to be used was made by psychiatrists on an individual basis, according to the needs of the patient. SSRI medication included fluvoxamine, 6 patients; fluoxetine 4; sertraline 3; and paroxetine, 2. Medium doses of SSRIs were used within the study in order to balance the effect of medication and potential side effects. Fluvoxamine and sertraline treatments were started at the dose of 50 mg once daily and increased to 150 mg once daily across a period of 6 weeks in 50 mg increments. The maximum dose of 150 mg daily was maintained until the end of the 12-week pharmacotherapy. Fluoxetine and paroxetine treatments were started at 20 mg/day and increased to 40 mg/day across a period of 4 weeks by 20 mg increments. The maximum dose of 40 mg/day was maintained until the end of the 12-week pharmacotherapy. We used the Asberg Rating Scale for Side Effects (SERS) to assess the side effects every 4 weeks. None of the patients dropped out or experienced severe side effects during SSRI treatment.

### MRI acquisition setting

We scanned OCD patients for DTI data before and after 12-week SSRI treatments. Healthy controls were scanned according to the same time frame as the patients at baseline. All data were acquired using a 1.5T magnetic resonance (MR) scanner (General Electric Medical Systems, Milwaukee, WI, USA). Axial T1-weighted imaging was conducted before the DTI scan in order to quickly locate obvious lesions of cerebral parenchyma. A total of 26 DTI volumes were acquired, including 25 volumes applied with diffusion gradient along 25 non-colinear directions (b = 1000 s/mm^2^), and one B0 volume without applying diffusion gradient (b = 0). For each volume, 24 continuous slices, each 5-mm-thick, were collected on a 24 cm×24 cm field of view, with the following imaging parameters: repetition time (TR) = 9000 ms, echo time (TE) = 105.5 ms, and matrix = 128*128. The acquisition time was 4 min 12 sec for each participant.

### Image processing

We adopted voxel-based analysis (VBA) in our study, which had been frequently used in previous OCD studies [6]–[12]. All data were processed using SPM8 (http://www.fil.ion.ucl.ac.uk/spm/), and FSL (http://www.fmrib.ox.ac.uk/fsl/) on a Linux based platform. The eddy current distortion was first corrected for each participant's DTI volume using the “eddycorrect” toolbox in FSL. Then, a brain mask, which separated brain and non-brain regions, was calculated from the B0 volume using the BET toolbox (FSL). Restricted within this mask, a diffusion tensor was fitted for each voxel in the brain image space using FSL's DTIFit. FA, AD, RD and MD maps were subsequently calculated.

Next, maps from the control group (23 participants) and patient group before medication (27 participants) were normalized to a B0-template for further statistical testing following a three-step procedure. An optimal method [30] was used, which is able to more closely match the subjects under investigation and the specific MRI acquisition setting in this study. Initially, a participant-specific B0 template was built with the data from the 23 controls. Each B0 volume was normalized to the SPM EPI template using SPM8's nonlinear coregistration method, with a reslicing resolution of 2×2×2 mm^3^. These normalized B0 volumes were then averaged and smoothed with a 6-mm FWHM Gaussian kernel to yield the B0 template. Thus the MRI acquisition setting of the resulting B0 template is the same as those data of each subject. Secondly, all original B0 volumes were again normalized to this B0 template, and the resulting transformation parameters applied to their corresponding FA, AD, RD and MD maps. Finally, following a popularly used schema - described in a previous study with OCD patients [9] - we smoothed all the transformed parameter maps with a 6-mm FWHM Gaussian kernel, ready for further statistical testing. In addition, because the study was focused on white matter abnormality of OCD patients, a white matter mask was created to restrict the search volume for statistics. We first generated a probabilistic tissue map of white matter from the template's B0 image using a tool incorporated in SPM, called “segment”. As the voxel intensity of the probabilistic map ranged from 0.0 to 1.0, we used 0.6 as a threshold to yield this binary white matter mask. The value 0.6 means that the probability of a voxel belonging to white matter is 60%, which we believe is an appropriate threshold to create the binary mask.

To compare the DTI-derived parameters of the patients before and after medication, FA, MD, AD and RD maps from the 15 patients after medication were also normalized to the standard space. To minimize the normalization error, each B0 volume of the post-medication patients was first coregisted to its corresponding B0 volume - collected from the same patient before medication - with the resulting transformation applied to its corresponding FA, AD, RD and MD maps. Then the transformation from patients' B0 volumes before medication to B0 template were applied to these coregisted parameter maps to bring them into the final B0-template space. Finally, a 6-mm FWHM Gaussian smoothing was performed on these normalized parameter maps.

### Statistics

We performed a whole-brain voxel-based analysis using the normalized and smoothed FA, AD, RD and MD maps for a 2-sample group t-test, comparing the 23 control participants and 27 patients before medication. Voxels of T>2.674 (p<0.001, uncorrected, cluster size>10 voxels; 2-tailed) were considered as indicating significant difference between the patient and control groups. This P value has been commonly used in previous DTI-based OCD studies [8], [12]. The white matter mask was used to confine the statistic only in the white matter region. Averaged maps were created from the normalized FA, AD, RD and MD data from all 50 participants, respectively. Then the corresponding t-test results were superimposed onto the averaged maps, using xjview (http://www.alivelearn.net/xjview8).

The mean FA values in regions which previously showed significant FA difference were calculated for control participants, patients before and patients after medication using the normalized and smoothed FA maps. Similar calculations were also made for AD, RD and MD. The values of these parameters were then correlated to the clinical scores using Pearson's partial correlation analyses, adjusted with age as a controlling factor. A General Linear Model repeated-measure analysis of covariance (ANOVA) was used in the analysis of each DTI-derived parameter before and after treatment. Pre and post-treatment and brain regions with abnormal DTI-derived parameters were set as within-subject factors. ANOVAs with least significant difference (LSD) post hoc tests were used to compare regional DTI-derived parameter changes before and after treatment in OCD patients. Independent t-tests were employed in the analyses of group differences in demographic variables between OCD patients and normal controls, and in demographic and clinical features between drug-naive and previously medicated OCD patients. Paired t-tests were used to compare clinical variables before and after treatment in OCD patients. The tests were two-tailed and the threshold of statistical significance was set at p<0.05.

## Results

### DTI-derived parameters at baseline

**Figure 1:**
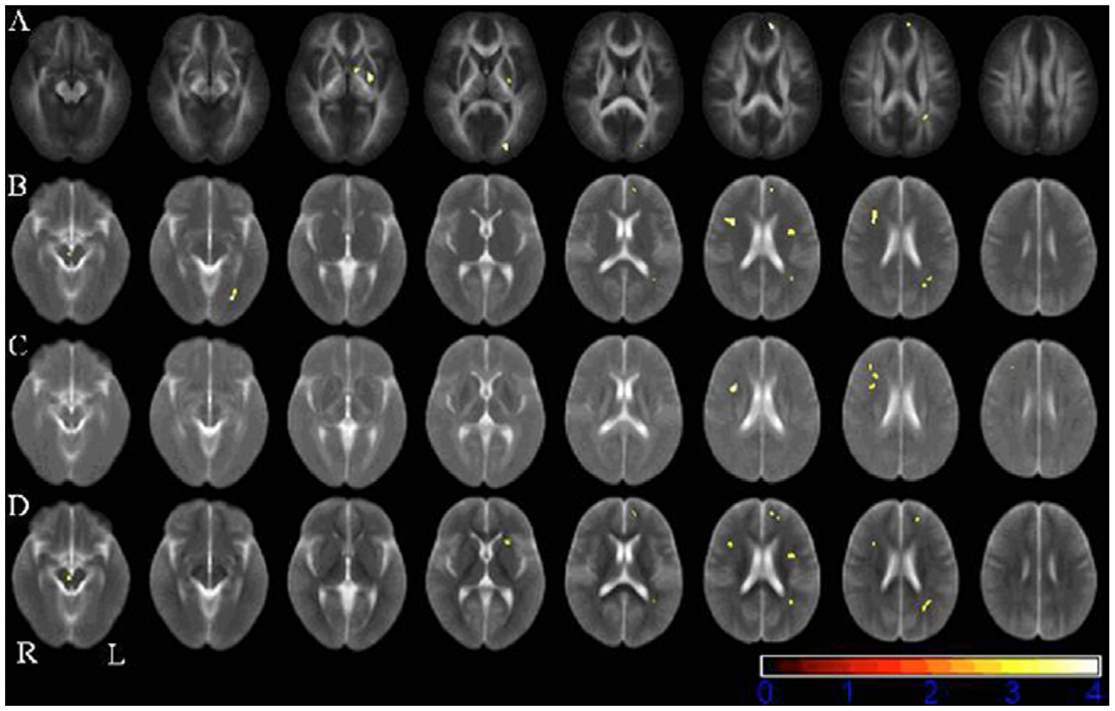
Voxel-based analysis between 27 patients with obsessive-compulsive disorder and 23 healthy controls before medication. The results were overlaid on mean images of corresponding diffusion tensor imaging (DTI) parameters. A. Fractional Anisotropy (FA) decrease; B. Mean Diffusion (MD) increase; C. Axial diffusion (AD) increase; D. Radial diffusion (RD) increase; p<0.001 uncorrected and cluster size>10 voxels. No increased FA and decreased diffusion coefficients (AD, RD and MD) were found.

The results indicate significantly decreased FA in patients compared with healthy controls in the left medial superior frontal gyrus, temporo-parietal lobe and middle occipital gyrus and around the left striatum white matter. Significantly higher RD in patients was shown in the same regions of the left medial superior frontal gyrus, temporal lobe, occipital lobe and striatum that investigated decreased FA. Three other brain areas - the left insula, right frontal lobe and midbrain - demonstrated increased RD and normal FA. Significantly higher AD in patients compared with healthy controls was observed only in the right frontal lobe, where RD was found to be higher while FA was normal. Accompanied by increased RD, there is significantly increased MD in OCD patients compared with healthy controls in the same brain areas of left medial superior frontal gyrus, temporal lobe, occipital lobe, insula and right frontal lobe and midbrain. These regions are illustrated in Figure 1. Montreal Neurological Institute (MNI) coordinates and cluster sizes of the findings are shown in Table 2. Significantly higher FA and lower RD, AD and MD were not shown in any part of the brain white matter in OCD patients compared with healthy subjects.

**Table 2:** Areas of abnormal DTI-derived parameters in 27 patients with obsessive-compulsive disorder and 23 healthy controls before medication.

Diffusion parameter	Region	Peak coordinates* (x,y,z)			Cluster size	t value
**FA**	Left medial superior frontal gyrus	−10	52	16	29	3.79
	Left striatum	−26	−6	−2	55	4.36
	Left temporo-parietal lobe	−28	−54	22	11	3.91
	Left middle occipital gyrus	−22	−88	6	31	4.53
**RD**	Left medial superior frontal gyrus	−10	50	16	27	4.50
	Left striatum	−24	16	4	13	3.87
	Left temporal lobe	−34	−52	18	29	3.77
	Left fusiform gyrus, occipital lobe	−26	−74	−6	12	3.59
	Left insula	−34	2	18	14	3.94
	Right frontal lobe	34	14	20	17	4.00
	Right midbrain	2	−26	−16	14	4.39
**AD**	Right frontal lobe	34	8	18	71	4.44
**MD**	Left medial superior frontal gyrus	−10	50	16	12	4.13
	Left temporal lobe	−28	−62	22	25	3.72
	Left fusiform gyrus, occipital lobe	−26	−76	−8	32	4.30
	Left insula	−34	2	18	12	3.78
	Right frontal lobe	34	14	20	61	4.77
	Right midbrain	2	−26	−16	10	4.10

* peak coordinateds represent the location of the maximum pixel values in standard Montreal Neurological Institute (MNI) space.

df = 48; p<0.001 (uncorrected).

To compare DTI-derived parameters of patients with and without a history of medication, mean FA, RD, AD and MD values were extracted in regions where significant differences had been found previously between the patient and healthy groups. We found no significant group difference in terms of mean FA, RD, AD and MD values (Table 3).

**Table 3:** Mean FA, RD, AD and MD values between drug-naive and previously medicated OCD patients.

Diffusion Parameter	Region	Drug-naïve patients (n = 13)	previously medicated patients (n = 14)	t	p
**FA**	Left medial superior frontal gyrus	0.200±0.015	0.195±0.018	0.099	0.922
	Left striatum	0.334±0.029	0.335±0.020	−0.142	0.888
	Left temporo-parietal lobe	0.315±0.024	0.305±0.026	0.986	0.334
	Left middle occipital gyrus	0.250±0.027	0.243±0.042	0.507	0.617
**RD**	Left medial superior frontal gyrus	0.756±0.038	0.744±0.061	0.599	0.554
	Left striatum	0.638±0.029	0.627±0.038	0.829	0.415
	Left temporal lobe	0.660±0.023	0.661±0.051	−0.048	0.962
	Left fusiform gyrus, occipital lobe	0.652±0.060	0.651±0.059	0.055	0.957
	Left insula	0.640±0.021	0.623±0.041	1.289	0.209
	Right frontal lobe	0.642±0.038	0.618±0.033	1.730	0.096
	Right midbrain	0.783±0.050	0.783±0.064	0.002	0.998
**AD**	Right frontal lobe	1.002±0.026	0.986±0.045	1.132	0.268
**MD**	Left medial superior frontal gyrus	0.850±0.034	0.837±0.061	0.666	0.512
	Left temporal lobe	0.800±0.022	0.800±0.053	−0.014	0.989
	Left fusiform gyrus, occipital lobe	0.830±0.058	0.820±0.063	0.418	0.680
	Left insula	0.754±0.022	0.739±0.043	1.089	0.287
	Right frontal lobe	0.759±0.032	0.740±0.030	1.611	0.120
	Right midbrain	0.943±0.053	0.946±0.067	−0.093	0.926

The units of radial diffusivity (RD), axial diffusivity (AD) and mean diffusivity (MD) is 10^−3^ mm^2^/s.

The total Y-BOCS scores (r = 0.40, df = 24, p = 0.04) and compulsive subscale scores (r = 0.50, df = 24, p = 0.01) were positively correlated with FA in the left striatum. None of the diffusion multi-parameters correlated significantly with HAMA, HAMD, age at onset or illness duration.

### Posttreatment changes in DTI-derived parameters

After 12 weeks of SSRI treatment, mean Y-BOCS scores, mean HAMA scores and mean HAMD scores in OCD patients were 9.9 (SD = 3.2), 6.4 (SD = 3.9) and 5.5 (SD = 2.6) respectively. This demonstrates significant improvements of 54% (SD = 16%) (t = 9.31, df = 14, p<0.001), 50% (32%) (t = 5.85, df = 14, p<0.001) and 45% (SD = 28%) (t = 4.34, df = 14, p = 0.001) respectively.

**Figure 2:**
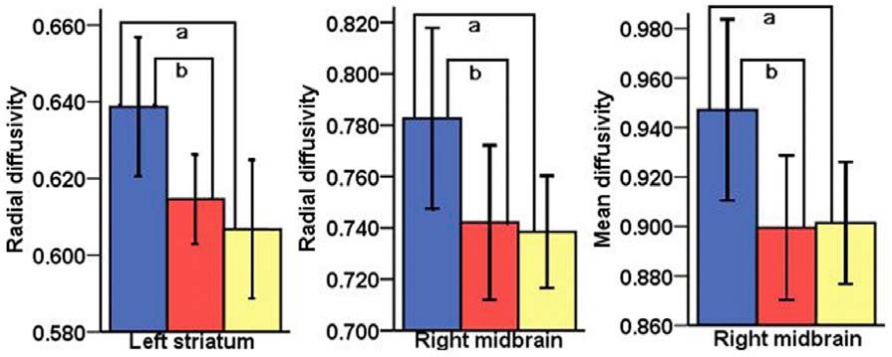
Regional radial diffusivity and mean diffusivity in follow-up patients before and after treatment and healthy controls. Blue bar represents follow-up patients at baseline (N = 15), red bar represents follow-up patients after treatment (N = 15), yellow bar represents healthy controls (N = 23). Error bars is 95% CI (confidence intervals). The units of radial diffusivity (RD) and mean diffusivity (MD) is 10^−3^ mm^2^/s. ^a^ Significant difference between obsessive-compulsive disorder patients and healthy controls (p<0.001, ANOVA with least significant difference post hoc test). ^b^ Significant difference between follow-up patients with obsessive-compulsive disorder before and after treatment (p<0.05, ANOVA with least significant difference post hoc test).

To evaluate DTI-derived parameter changes in patients before and after 12-week SSRI treatment, mean FA, RD, AD and MD values were extracted in regions where significant differences had previously been found between the patient and healthy groups. The effect of pre and post-treatment in brain regions with abnormal RD values was found to be statistically significant (F = 7.079, df = 1,14, p = 0.019). There was a significant pre and post-treatment × brain regions with abnormal RD values interaction (F = 5.375, df = 4,54, p = 0.001). Although the effect of pre and post-treatment in the brain regions with abnormal MD values was not statistically significant (F = 3.669, df = 1,14, p = 0.076), the pre and post-treatment × brain regions with abnormal MD values interaction was found to be significant (F = 6.869, df = 3,43, p = 0.001). As to the regional differences, decreased RD was observed in the left striatum (baseline = 0.639×10^−3^ mm^2^/s (SD = 0.033), follow-up = 0.615×10^−3^ mm^2^/s (SD = 0.021), p = 0.026) and right midbrain (baseline = 0.783×10^−3^ mm^2^/s (SD = 0.064), follow-up = 0.742×10^−3^ mm^2^/s (SD = 0.054), p = 0.044). Decreased MD was also found in the right midbrain (baseline = 0.947×10^−3^ mm^2^/s (SD = 0.066), follow-up = 0.900×10^−3^ mm^2^/s (SD = 0.053), p = 0.019) (Figure 2). No significant changes in DTI-derived parameters were found in the left medial superior frontal gyrus, temporal lobe, occipital lobe, insula and right frontal lobe (Table S1).

In order to reduce the possibility of potential type I errors caused by multiple comparisons, we analyzed correlations between symptom changes and DTI derived parameters, which were greatly changed before and after treatment. Decreases in Y-BOCS compulsive subscale scores were significantly correlated with the RD (r = −0.71, df = 12, p = 0.01) values in the left striatum, RD (r = −0.57, df = 12, p = 0.03) and MD (r = −0.58, df = 12, p = 0.03) in the right midbrain at baseline. The mean percentage of RD in the left striatum changed by 4% and RD and MD in the right midbrain both changed by 5%. However, no significant correlations were found either between percentage changes in clinical symptoms and percentage changes in DTI-derived parameters, or between baseline clinical symptoms and percentage changes in DTI-derived parameters.

## Discussion

This is the first DTI study using both multiple parameters and unmedicated OCD patients. This study was therefore designed to be exploratory, using a whole-brain analysis to provide a broad perspective of the pathology found in this patient sample. Two main findings emerged from this study. First, OCD patients demonstrated abnormalities of white matter microstructure, particularly in terms of myelin integrity in multiple sites of the fronto-striato-thalamo-cortical loop. Second, these abnormalities were not a result of previous medication use. Indeed, there are indications that some abnormalities could be partly reversed by SSRI treatment. The interesting findings emerging from this study contribute to the identification of regions of interest relevant to OCD research. At the same time, the data indicates possible regions of focus (e.g. myelination integrity), which may be relevant to ongoing research.

In the present study, abnormal DTI-derived parameters in OCD patients were observed in the white matter including the left medial superior frontal gyrus, temporo-parietal lobe, occipital lobe, insula, right frontal lobe and midbrain and white matter around the left striatum. These structures are located within important brain regions relevant to the pathophysiology of OCD, and indicate abnormalities primarily in the fronto-striato-thalamo-cortical loop. Baseline DTI-derived parameters did not differ between patients with and without medication history.

The prefrontal cortex is primarily involved in cognitive function and emotional information processing in human beings. Both structural and functional imaging studies have supported the existence of abnormalities within the prefrontal cortex, particularly the orbitofrontal cortex, in OCD patients [3], [4], [31]. However, two studies reported higher FA and ADC in the medial frontal region [6], [7], which is inconsistent with our findings. In the study of Menzies et al. [6], 23 of the 30 OCD patients had been prescribed the SSRIs, clomipramine and quetiapine. In another study, 2 of 15 OCD patients had comorbid mood disorders and all patients were taking drug treatments for OCD [7]. Yoo et al. have noted in their study that white matter alterations may be partly reversible with citalopram treatment [12]. Therefore, differences in the patient sample may account in part for this inconsistency.

More and more evidence demonstrates that other cortical structures, beside the frontal lobe, participate in the frontal-subcortical circuit [32]. The temporo-parietal junction is a complex sensory cortical area, which is involved in perception and awareness. The fusiform gyrus is thought to be an occipito-temporal gyrus. The fusiform gyrus and the occipital lobe are involved in visual processing, face and body recognition, and word identification. Koprivova et al. [33] found total white matter volume reduction, with marked reduction particularly in the right temporo-parieto-occipital and left middle temporal gray matter in OCD patients. Furthermore, functional magnetic resonance imaging studies have shown abnormalities in the temporal lobe, parietal lobe and occipito-temporal region [34], [35]. Similar findings have been reported by Szeszko et al. [8] and Menzies et al. [6], suggesting that OCD patients possess lower FA in the bilateral parietal and left occipital lobe. No other DTI studies have reported temporal lobe abnormalities in OCD. The insular has several functions, including interoceptive awareness, motor control, homeostasis and emotional functioning. These functions are all related to the symptoms of OCD. Pujol et al. [36] reported that their sample of 72 OCD patients demonstrated reduced gray matter volume in the medial frontal gyrus, the medial orbitofrontal cortex and the left insulo-opercular region. One PET study indicated a reduction of serotonin transporter binding in the insular cortex in OCD patients [37]. Our study also found that myelin integrity was damaged in the insular cortex in OCD subjects.

The striatum forms a key region within the subcortical structures involved in the pathophysiology of OCD, and is mainly involved in the planning and modulation of movement pathways. In contrast to our study, two studies have shown significantly higher FA in the internal capsule and white matter in the area superolateral to the right caudate among OCD patients [11], [12]. The small sample size of these two studies may explain their inconsistency with the results of our study. We also found that FA variables were positively correlated with the severity of compulsive symptoms in the left striatum. Ha et al. [9] also showed a positive correlation between the Y-BOCS obsessive subscale scores and mean FA values, with the suggestion that this relationship emerges as a compensatory mechanism for neuronal alternations in the anterior cingulate. A further two MRI studies have reported a relationship between striatum volumes and severity of OCD symptoms [36], [38]. Our results support the proposal that the striatum may be involved in the compulsive symptoms of OCD.

The neurons of the raphe nuclei are distributed along the brainstem and midbrain and are closely related with the serotonin system. Metabolic imaging studies including PET and SPECT have shown decreased serotonin transporter availability in the midbrain and brainstem of OCD patients [39]–[41]. In our study, abnormal white matter microstructure was found in the right midbrain of OCD patients as compared to healthy controls. No other DTI studies have found midbrain abnormalities in OCD. Further studies should be undertaken to investigate the relationship between myelin integrity abnormalities and the serotonin system in OCD patients.

The pathophysiological processes of decreased FA and/or increased MD in OCD patients are indicative of white matter microstructural abnormalities. Higher RD in combination with no change in AD among OCD patients was found in the left medial superior frontal gyrus, temporo-parietal lobe, occipital lobe, striatum, insula and right midbrain, further suggesting that disruptions of myelin integrity contributed to the white matter abnormalities in these regions. This finding is supported by recent genetic studies. There was biased transmission of polymorphisms in the genes involved in myelination in OCD, such as myelin oligodendrocyte glycoprotein (MOG) [42] and oligodendrocyte lineage transcription factor 2 (OLG2) [43]. Atmaca et al. provided further evidence that the MOG G511C (Val142Leu) polymorphism might be associated with changes in the total white matter volume of OCD patients [44]. The results of these studies support the existence of structural abnormalities of myelin in OCD. We noticed higher AD along with higher RD and MD, and normal FA in the right frontal lobe, which suggests a pattern of both myelination and axonal deficits. A possible explanation is that axial diffusivity occurs as an effective compensatory response when the myelin integrity is impaired.

We also found that abnormal myelin integrity in the left striatum and right midbrain of OCD patients was reversible by 12-week SSRI therapy. Several imaging studies support the finding that abnormalities of frontal-subcortical circuits in OCD patients are partly improved after treatment [45]–[51]. In particular, decreased activation within the left putamen [49] and increased dysfunctional availability of serotonin transporter in the midbrain [51] were found after medication in two studies. Yoo et al. further identified that the white matter changes in the FA variable were partly reversible in the right posterior thalamic radiation after 12 weeks of citalopram treatment in OCD patients [12]. There is no direct study exploring the potential pharmacological roles of SSRI on myelination. However, SSRIs are considered to have the effect of neuroprotection and promote activation of Brain Derived Neurotrophic Factor (BDNF) [52]. Xiao et al. proved that BDNF could directly effect oligodendrocytes and increase central nervous system myelination [53]. Interestingly, although we did not find correlations between changes in DTI-derived values and clinical symptoms, DTI-derived parameters before SSRI treatment were significantly correlated with changes in compulsive symptoms. Our findings suggest that baseline DTI-derived parameters in several regions including the left striatum, right frontal lobe and midbrain may be related to the clinical efficacy of SSRI.

Please note although the statistics conducted in this study was uncorrected using a cluster of 10 voxels, we used a cruel p-value threshold of <0.001 to indicate statistical confidence. The use of a small cluster size is in part due to the quality of DTI data collected within this preliminary study. Further research based on this preliminary study and using DTI data of improved image quality, should be driven by carefully designed hypotheses with more rigorous statistical standards.

Several other limitations also exist in the present study. One problem for voxel-based analysis (VBA) is related to multiple comparisons and, therefore, the potential for type I error. To limit the impact of this on the final results, we used RD, AD and MD as complementary measures to the FA result. Another major shortcoming is that VBA is unable to directly provide indications of abnormal connectivity between anatomical structures. Fiber tracking techniques may present an appropriate option to deal with this problem, and will form an aspect of future work following this paper. Slight differences among the four SSRI drugs used in the present study should be considered, although they are all variations of SSRIs and share common pharmacological mechanisms. There is a possibility that changes in DTI-derived parameters in several brain regions before and after 12-week SSRI treatment were related to the passage of time. Further studies employing a “placebo-treated group” would help to tackle this query.

In conclusion, our preliminary findings suggest that in OCD abnormalities of white matter microstructure, particularly in terms of myelin integrity, primarily affect the fronto-striato-thalamo-cortical circuit, independent of previous medication history. These abnormalities attest to the role of white matter in the pathophysiology of OCD. Some abnormalities may be partly reversed by SSRI treatment.

## Supporting Information

Table S1 **Regional differences in FA, RD, AD and MD before and after treatment in OCD patients.**
(DOC)
