# Supplementary material for: Abnormalities of White Matter Microstructure in Unmedicated Obsessive-Compulsive Disorder and Changes after Medication
Source: PLoS One. 2012 Apr 27;7(4):e35889. doi: 10.1371/journal.pone.0035889 (PMC3338776; doi:10.1371/journal.pone.0035889)
Supplement: Table S1 — Regional differences in FA, RD, AD and MD before and after treatment in OCD patients. (DOC) [file pone.0035889.s001.doc]

| **Diffusion**  **parameter** | **Region** | Patients group before treatment  (n=15) | Patients group after treatment  (n=15) | Healthy controls  (n=23) | p |
| --- | --- | --- | --- | --- | --- |
| **FA** | Left medial superior frontal gyrus | 0.191±0.017 | 0.201±0.023 | 0.216±0.018 | 0.430 |
|  | Left striatum | 0.330±0.022 | 0.345±0.026 | 0.359±0.017 | 0.222 |
|  | Left temporo-parietal lobe | 0.307±0.025 | 0.305±0.031 | 0.336±0.024 | 0.894 |
|  | Left middle occipital gyrus | 0.252±0.030 | 0.255±0.034 | 0.286±0.022 | 0.851 |
| **RD** | Left medial superior frontal gyrus | 0.746±0.052 | 0.730±0.049 | 0.701±0.038 | 0.358 |
|  | Left striatum | 0.639±0.033 | 0.615±0.021 | 0.603±0.030 | 0.026 |
|  | Left temporal lobe | 0.668±0.043 | 0.657±0.038 | 0.622±0.033 | 0.396 |
|  | Left fusiform gyrus, occipital lobe | 0.660±0.065 | 0.655±0.065 | 0.610±0.027 | 0.798 |
|  | Left insula | 0.633±0.037 | 0.636±0.028 | 0.599±0.026 | 0.782 |
|  | Right frontal lobe | 0.641±0.041 | 0.630±0.042 | 0.596±0.030 | 0.420 |
|  | Right midbrain | 0.783±0.064 | 0.742±0.054 | 0.730±0.047 | 0.044 |
| **AD** | Right frontal lobe | 0.995±0.041 | 0.993±0.022 | 0.956±0.032 | 0.879 |
| **MD** | Left medial superior frontal gyrus | 0.837±0.052 | 0.826±0.042 | 0.799±0.040 | 0.492 |
|  | Left temporal lobe | 0.807±0.045 | 0.796±0.039 | 0.766±0.029 | 0.409 |
|  | Left fusiform gyrus, occipital lobe | 0.829±0.070 | 0.830±0.068 | 0.780±0.030 | 0.958 |
|  | Left insula | 0.746±0.040 | 0.750±0.022 | 0.715±0.028 | 0.710 |
|  | Right frontal lobe | 0.759±0.035 | 0.748±0.028 | 0.718±0.023 | 0.317 |
|  | Right midbrain | 0.947±0.066 | 0.900±0.053 | 0.894±0.046 | 0.019 |
| Abbreviations: FA, fractional anisotropy; RD, radial diffusivity; AD, axial diffusivity; MD, mean diffusion.  The units of radial diffusivity (RD), axial diffusivity (AD) and mean diffusivity (MD) is 10^-3^ mm^2^/s.  p value represents the comparisons of DTI-derived parameters before and after treatments using analyses of variance (ANOVAs) with least significant difference (LSD) post hoc tests. | | | | | |
